# Vitamin D and Sunlight Exposure in Newly-Diagnosed Parkinson’s Disease

**DOI:** 10.3390/nu8030142

**Published:** 2016-03-04

**Authors:** Juan Wang, Deyu Yang, Yu Yu, Gaohai Shao, Qunbo Wang

**Affiliations:** 1 Department of Neurology, Yongchuan Hospital of Chongqing Medical University, Chongqing 404100, China; wxsyzc@yeah.net (J.W.); wakeupinfall@126.com (D.Y.); 2 Department of Orthopedics, Yongchuan Hospital of Chongqing Medical University, Chongqing 404100, China; shaogaohai567@163.com (G.S.); wqb631113@163.com (Q.W.)

**Keywords:** Parkinson’s disease, sunlight exposure, vitamin D

## Abstract

Circulating vitamin D has previously been found to be lower in patients with Parkinson’s disease (PD), while the effects of sunlight exposure have not yet been fully investigated. Therefore, we evaluated the associations between serum vitamin D, vitamin D intake, sunlight exposure, and newly-diagnosed PD patients in a Chinese population. This case-control study measured serum 25-hydroxyvitamin D (25(OH)D) levels and sunlight exposure in 201 patients with newly-diagnosed PD and 199 controls without neurodegenerative diseases. Data on vitamin D intake and sunlight exposure were obtained using a self-report questionnaire. Multivariable logistic regressions were employed to evaluate the associations between serum 25(OH)D levels, sunlight exposure, and PD. Adjustments were made for sex, age, smoking, alcohol use, education, BMI, and vitamin D intake. There were significantly lower levels of serum 25(OH)D (20.6 ± 6.5 ng/mL), daily vitamin D intake (8.3 ± 3.7 g/day), and sunlight exposure (9.7 ± 4.1 h/week) in patients with PD compared to healthy controls (*p* < 0.05). Crude odds ratios (ORs) for PD in the quartiles of serum 25(OH)D were 1 (reference), 0.710 (0.401, 1.257), 0.631 (0.348, 1.209), and 0.483 (0.267, 0.874), respectively. Crude ORs for PD in quartiles of sunlight exposure were 1 (reference), 0.809 (0.454, 1.443), 0.623 (0.345, 1.124) and 0.533 (0.294, 0.966), respectively. A significant positive correlation between serum 25(OH)D and sunlight exposure was found, but serum 25(OH)D was not correlated with daily vitamin D intake. This study indicates that lower levels of serum 25(OH)D and sunlight exposure are significantly associated with an increased risk for PD.

## 1. Introduction

Parkinson’s disease (PD) is a common progressive neurodegenerative disorder mostly occurring in elderly humans. Although the pathogenesis of PD is unclear, environmental factors, including some vitamins, have been suggested to be linked with the development of PD [1,2,3,4,5,6].

Previously, vitamin D was considered to be only related to bone metabolism, osteoporosis, and falling. However, vitamin D has recently emerged as a critical factor in the pathogenesis of chronic diseases and progressive neurodegenerative disorders [5,7,8]. Additionally, inadequate amounts of vitamin D may play a role in the development of PD [9], as the loss of dopaminergic neurons in the brain of PD patients could be related to a condition of continuously inadequate vitamin D [7].

Low circulating vitamin D occurs in populations of all ages and geographic latitudes. Sources of vitamin D include exposure to sunlight, diet, and vitamin D supplements. However, neither diet alone, nor the current recommendation of 800 to 1200 international units of vitamin D supplementation may be enough to provide adequate levels of circulating 25(OH)D. Moreover, 20% of vitamin D is obtained from food (eggs, fish, animal liver, and dairy products), whereas 80% is obtained from ultraviolet radiation inducing skin synthesis as a product of skin 7-dehydrocholesterol trans-formations [10]. Previous studies indicated that sunlight exposure could increase bone mineral density by increasing serum 25-OHD, thereby preventing hip fracture [11,12,13]. Therefore, both circulating vitamin D and sunlight exposure should be considered when investigating association between PD and vitamin D, but epidemiological data remain limited. In this study, we measured serum 25(OH)D, sunlight exposure, and vitamin D intake, and evaluated their potential relationship with PD risk in a Chinese population.

## 2. Methods

A total of 201 newly-diagnosed cases of PD were recruited from the clinic of the Department of Neurology, Yongchuan Hospital of Chongqing Medical University, Chongqing, China, while 199 age- and sex-matched healthy controls were recruited from three community hospitals during the winter of 1 November 2014 and 1 January 2015 in Chongqing, China. PD was diagnosed by neurologists according to the UK PD Society Brain Bank clinical diagnostic criteria. Participants were excluded if they fulfilled any of the following exclusion criteria: presence of any other neurodegenerative disease, thyroid dysfunction, and recent modification of lifestyles or dietary patterns. All participants completed a questionnaire-based interview and received a physical examination. Interviewers, doctors, and nurses were trained in administering the questionnaire and physical examination prior to study commencement. All participants were informed about the details of the study and signed a consent form. The study protocol was approved by the local ethics committee.

Educational status, sunlight exposure habit, smoking, drinking, and medical history of all participants were obtained by face-to-face questionnaires. Dietary intake was measured by the validated semi-quantitative food-frequency questionnaire [14]. Required information on the use of vitamin D supplements included time and dose of the supplements. Total vitamin D intake was calculated as dietary vitamin D intake plus supplemented vitamin D. Dietary vitamin D intake was calculated using the Food Nutrition Calculator (V1.60; Chinese CDC). Body mass index (BMI) was calculated as weight (kg) divided by the square of height (m).

After fasting for 10 h, samples of blood were obtained from all participants, centrifuged at 2500 g for 15 min for serum, and stored at −80 °C. Serum 25(OH)D concentrations were detected by ACQUITY Ultra Performance Liquid Chromatography (Waters, Milford, MA, USA). Inter- and intra-assay coefficients of 25(OH)D at 20 ng/mL were 4.56% and 3.81%, respectively. Serum 25(OH)D concentration levels of less than 20 ng/mL were considered as vitamin D deficiency; levels between 20 and 30 ng/mL were considered as vitamin D insufficiency; and levels over 30 ng/mL were considered as vitamin D sufficiency [2].

### Statistical Analysis

Clinical variables were expressed as mean ± standard deviation (SD) or percentages as appropriate. The chi-square test was employed to assess the differences in proportions. Associations between serum 25(OH)D, sunlight exposure and PD development were analyzed by multiple variable logistic regression analysis by adjusting for the covariates of age, sex, BMI, smoking, alcohol use, and vitamin D intake. The correlations between serum 25(OH)D and sunlight exposure were analyzed by multiple linear regression analysis. A value of *p* < 0.05 was considered statistically significant.

## 3. Results

### 3.1. Serum 25(OH)D and Sunlight Exposure in Participants

Table 1 presents the characteristics of the participants. There were no significant differences of age, sex, BMI, education, proportion of smoking, alcohol use, vitamin D supplements, and status between PD patients and the healthy control group. Compared with healthy control subjects, patients with PD had significantly lower levels of serum 25(OH)D, vitamin D intake, and sunlight exposure hours per week.

### 3.2. Associations between Serum 25(OH)D, Sunlight Exposure, and PD

As serum 25(OH)D was not correlated with vitamin D intake, vitamin D intake was analyzed as a covariate in the logistic regression (Table 2). 25(OH)D and sunlight exposure was analyzed in the same model. In the crude analysis, serum 25(OH)D and sunlight exposure were both inversely associated with the risk of PD. Serum 25(OH)D was categorized in quartiles: 1 = 0–18 ng/mL, 2 = 18–22 ng/mL, 3 = 22–25 μg, and 4 = >25 ng/mL. Sunlight exposure was also grouped in quartiles: 1 = 0–7 h, 2 = 7–10 h, 3 = 10–14 h, and 4 = >14 h per week. A negative association between serum 25(OH)D and PD was observed at the top quartile. When adjusted for age, sex, alcohol use, smoking, education, BMI, sunlight exposure, and vitamin D intake, adjusted odds ratios (ORs) for PD in the quartiles of serum 25(OH)D intake were 1 (reference), 0.668 (0.373, 1.197), 0.656 (0.356, 1.209), and 0.499 (0.268, 0.930), respectively. The adjusted ORs for PD in the quartiles of sunlight exposure were 1 (reference), 0.814 (0.447, 1.482), 0.623 (0.340, 1.141) and 0.506 (0.274, 0.932), respectively, after adjustment for age, sex, alcohol use, smoking, education, BMI, serum 25(OH)D, and total vitamin D intake.

### 3.3. Correlations between Serum 25(OH)D and Sunlight Exposure

**Figure 1:**
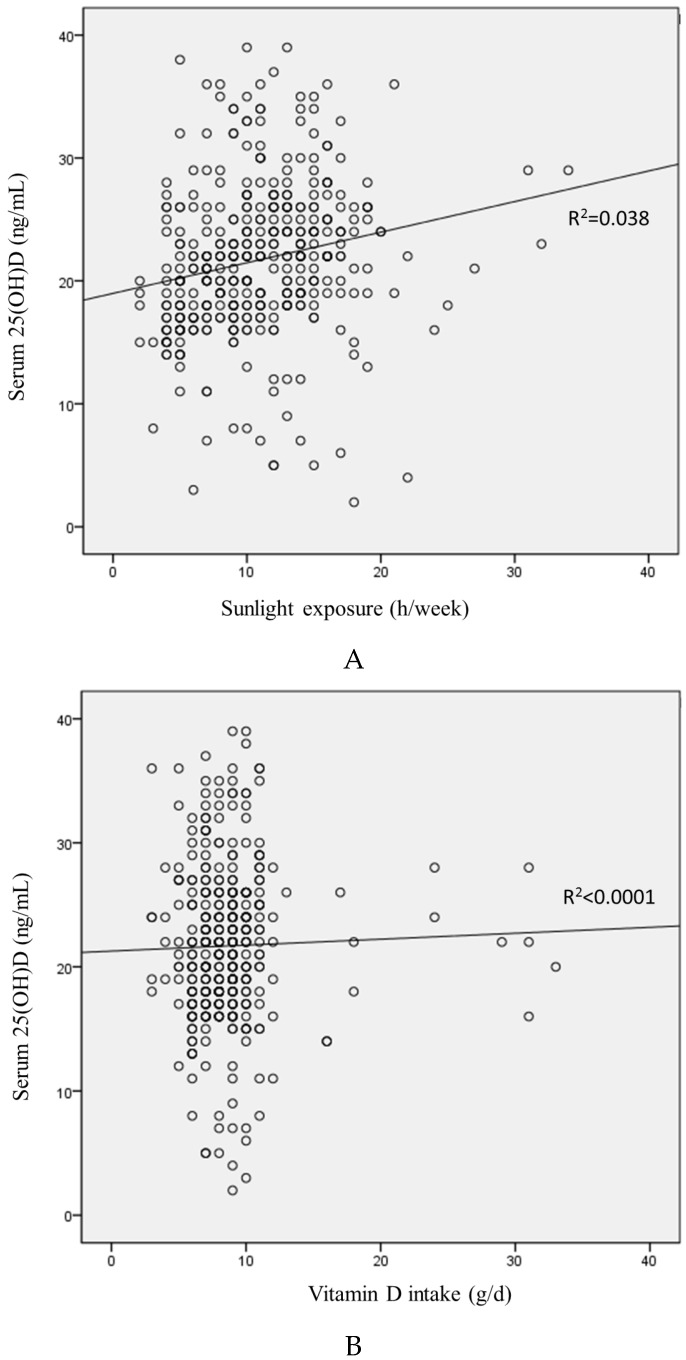
The plots of serum 25(OH)D, sunlight exposure (**A**) and dietary vitamin D (**B**).

A significant positive correlation between serum 25(OH)D and sunlight exposure was observed, but serum 25(OH)D was not correlated with daily vitamin D intake (Figure 1). Table 3 presents the associations between serum 25(OH)D and sunlight exposure in groups, which were further evaluated by multivariate linear regression analysis in different models. Serum 25(OH)D remained positively associated with sunlight exposure after multiple adjustments (all *p* < 0.05).

## 4. Discussion

In this case-control study, we found that levels of serum 25(OH)D and sunlight exposure were lower in patients with PD and were inversely associated with the risk for PD. Several studies have indicated lower levels of serum vitamin D in PD [7,9] but lack dietary intake or sunlight exposure measures. Moreover, a longer duration of PD may affect the results in these studies. To the best of our knowledge, this is the first case-control study designed to evaluate the associations between serum 25(OH)D, vitamin D intake, sunlight exposure, and newly diagnosed PD in a relatively large-scale Chinese population.

The present study supports the hypothesis that lower serum vitamin D could be a risk factor for PD. Although it is unclear how vitamin D could protect against PD, vitamin D has been found necessary for the regulation of some neurodegenerative processes, such as neurotrophin, inducible nitric oxide synthase, glutathione and monoamine synthesis, and apoptosis. In addition, vitamin D has multiple beneficial effects on the nervous system, such as anti-oxidative stress, calcium regulation of neurons, immune system modulation, nerve conduction strength, and detoxification [15]. Additionally, vitamin D supplementation could cause a decrease in lipid peroxidation and enhance SOD activity, an antioxidant in the liver, and reverse impaired liver metabolism by decreasing free radicals in diabetic rats [8]. On a different note, elevated levels of vitamin D receptors and an enzyme responsible for the formation of the active form 1,25(OH)2D3 were observed in the substantia nigra, the brain region mostly affected by PD. Moreover, vitamin D3 supplementation may stabilize PD for a short period in patients with specific genotypes [16]. Current evidence suggested the possibility that chronic inadequacy of vitamin D may lead to the loss of dopaminergic neurons in the substantia nigra region and, subsequently, to the development of PD [17]. However, associations with dietary vitamin D have not been confirmed in population studies yet [5,18]. Small sample size, unmatched controls, and changed living habits might cover the mild effects of vitamin D intake on PD risk. However, we did not find a significant correlation between vitamin D intake and serum 25(OH)D, although total vitamin D intake was lower in the PD group. One possible explanation is that sunlight exposure might decrease the contribution of total vitamin D intake to serum 25(OH)D.

In order to reach conclusive results, future studies investigating the effects of sunlight exposure on serum 25(OH)D need to include the exposure time as a measurement. We found a significant inverse association between sunlight exposure and PD. In line with our study, Kenborg *et al.* reported that men working outdoors have a lower risk of PD [19]. However, to the best of our knowledge, no further studies have focused on sunlight exposure in a PD population. Serum vitamin D can be obtained from the diet (20%, e.g., from eggs, fish, animal liver, and dairy products) or is photosynthesized in the skin by the action of solar ultraviolet B radiation as a product of skin 7-dehydrocholesterol transformations (80%) [20]. Meanwhile, more frequent sunlight exposure along with increased physical activity could be another potential explanation for the protective role of vitamin D for PD. Greater intensity of physical activity was associated with faster completion of the Trail Making Test, higher levels of verbal fluency, higher brain volume, more white matter, and higher parietal lobe gray matter volume, situated bilaterally at the precuneus in elderly people. Thus, sunlight exposure might have multiple roles in the protection of PD. A high prevalence of vitamin D deficiency (50%–80%) has been reported in many parts of China [21,22,23], and low levels of dietary vitamin D intake and sun exposure might be potential underlying factors. Additionally, the rate of vitamin D supplementation was lower in the general population. However, no reliable study has investigated this public health problem yet.

Some limitations of the present study need to be considered. First, the design of a case-control study could not provide causal effects of serum vitamin D on PD development. Second, the vitamin D status could not be fully represented due to absent measurements of serum 1,25(OH)2D3. In addition, the study took place in winter and no measurements were taken during summer. Although serum vitamin D is relatively stable over time [24], the possibility of changed 25(OH)D and 1,25(OH)2D3 levels cannot be excluded [25]. Third, physical activity might interact with the vitamin D status in the development of PD, but this was not measured by this study. Future studies should consider including such parameters to reach more conclusive results.

## 5. Conclusions

In sum, our study found that lower levels of serum 25(OH)D and sunlight exposure are significantly associated with a higher risk for PD. Further cohort and vitamin D supplementation studies are needed to confirm this finding.

## Figures and Tables

**Table 1:** Demographic and clinical characteristics of the participants in this study.

Variables	Cases (*n* = 201)	Controls (*n* = 199)	*p* Values
age (year)	65.1 ± 8.8	64.1 ± 8.7	0.16
Sex (male/female)	64/137	77/122	0.09
Smoking, *N* (%)	43 (21.4)	53 (26.7)	0.13
Alcohol use, *N* (%)	55 (24.4)	52 (26.1)	0.43
Education, *N* (%)			
<9 years	88 (43.8)	76 (38.1)	0.39
9–12 years	66 (32.8)	74 (37.2)	
>12 years	47 (23.4)	49 (24.7)	
BMI (kg/m^2^)	24.5 ± 2.5	24.2 ± 2.3	0.32
Sunlight exposure (h/week)	9.7 ± 4.1	12.1 ± 5.2	<0.01
Vitamin D intake (g/day)	8.3 ± 3.7	9.0 ± 4.6	<0.01
Vitamin D supplements, *N* (%)	5(2.5)	3 (1.5)	0.72
Serum 25(OH)D (ng/mL)	20.6 ± 6.5	22.8 ± 5.5	0.04
Vitamin D insufficiency, *N* (%)	183 (91.0)	179 (89.9)	0.74
Vitamin D sufficiency, *N* (%)	18 (9.0)	20 (10.0)	

Differences between cases and controls were assessed using a *t*-test for continuous data and χ^2^ test for binary data. BMI: body mass index; *N*: number of participants. All variables are presented as mean ± SD or percentages as appropriate.

**Table 2:** Odds ratios (ORs) and 95% confidence intervals (CIs) for Parkinson’s disease by quartiles of serum vitamin D intake and sunlight exposure.

Variable	Quartile				*p* for Trend
	1 (Reference)	2	3	4	
Serum 25(OH)D					
Cases/Controls	63/38	53/49	45/52	40/60	
Crude OR (95% CI)	1	0.710 (0.401,1.257)	0.631 (0.348,1.146)	0.483 (0.267,0.874)	0.117
Adjusted OR^1^ (95% CI)	1	0.695 (0.390,1.238)	0.630 (0.345,1.150)	0.498 (0.269,0.919)	0.126
Adjusted OR^2^ (95% CI)	1	0.669 (0.374,1.197)	0.644 (0.350,1.184)	0.493 (0.265,0.918)	0.164
Adjusted OR^3^ (95% CI)	1	0.668(0.373,1.197)	0.656 (0.356,1.209)	0.499 (0.268,0.930)	0.181
Sunlight exposure					
Cases/Controls	62/39	53/46	45/55	41/59	
Crude OR (95% CI)	1	0.809 (0.454,1.443)	0.623 (0.345,1.124)	0.533 (0.294,0.966)	0.170
Adjusted OR^1^ (95% CI)	1	0.827 (0.459,1.489)	0.637 (0.350,1.160)	0.529 (0.290,0.965)	0.171
Adjusted OR^2^ (95% CI)	1	0.833 (0.459,1.512)	0.621 (0.340,1.135)	0.503 (0.273,0.926)	0.167
Adjusted OR^3^ (95% CI)	1	0.814 (0.447,1.482)	0.623 (0.340,1.141)	0.506 (0.274,0.932)	0.142

Adjusted OR^1^: adjusted for sex, age, and BMI; Adjusted OR^2^: adjusted for sex, age, BMI, smoking, alcohol use, and education; Adjusted OR^3^: adjusted for adjusted for sex, age, BMI, smoking, alcohol use, education, and vitamin D intake.

**Table 3:** The β-Coefficients of serum 25(OH)D and sunlight exposure in the multiple linear regression model.

Groups	Overall	*p*	Case	*p*	Control	*p*
Model 1	0.249(0.126–0.372)	<0.001	0.253(0.034–0.472)	0.024	0.175(0.028–0.323)	0.02
Model 2	0.237(0.116–0.358)	<0.001	0.245(0.017–0.472)	0.035	0.173(0.027–0.319)	0.02
Model 3	0.252(0.131–0.373)	<0.001	0.271(0.041–0.501)	0.021	0.178(0.032–0.323)	0.017
Model 4	0.252(0.131–0.373)	<0.001	0.234(0.035–0.480)	0.04	0.176(0.030–0.322)	0.018

Model 1: no adjustment; Model 2: adjusted for age, sex, and BMI; Model 3: further adjusted for smoking, alcohol use, and education; Model 4: further adjusted for vitamin D intake.
